# Zika Virus-Induction of the Suppressor of Cytokine Signaling 1/3 Contributes to the Modulation of Viral Replication

**DOI:** 10.3390/pathogens9030163

**Published:** 2020-02-27

**Authors:** Rak-Kyun Seong, Jae Kyung Lee, Ok Sarah Shin

**Affiliations:** Bk21 PLUS Program, Department of Biomedical Sciences, College of Medicine, Korea University Guro Hospital, Seoul 08308, Korea; cyeano66@naver.com (R.-K.S.); jae.lee0321@gmail.com (J.K.L.)

**Keywords:** suppressor of cytokine signaling, zika virus, interferon, antiviral

## Abstract

Zika virus (ZIKV) is a mosquito-borne flavivirus that has emerged and caused global outbreaks since 2007. Although ZIKV proteins have been shown to suppress early anti-viral innate immune responses, little is known about the exact mechanisms. This study demonstrates that infection with either the African or Asian lineage of ZIKV leads to a modulated expression of suppressor of cytokine signaling (SOCS) genes encoding SOCS1 and SOCS3 in the following cell models: A549 human lung adenocarcinoma cells; JAr human choriocarcinoma cells; human neural progenitor cells. Studies of viral gene expression in response to SOCS1 or SOCS3 demonstrated that the knockdown of these SOCS proteins inhibited viral NS5 or ZIKV RNA expression, whereas overexpression resulted in an increased expression. Moreover, the overexpression of SOCS1 or SOCS3 inhibited the retinoic acid-inducible gene-I-like receptor-mediated activation of both type I and III interferon pathways. These results imply that SOCS upregulation following ZIKV infection modulates viral replication, possibly via the regulation of anti-viral innate immune responses.

## 1. Introduction

First isolated in 1947, Zika virus infection typically manifests in mild clinical symptoms [1]. The virus, however, has recently emerged as an alarming threat to public health due to its potential to spread rapidly and be transmitted sexually and vertically, in addition to its association with congenital abnormalities and neurological complications in infected fetuses and adults [2,3,4,5]. Since 2015, many studies have applied diverse approaches to gain insight into the pathogenesis and underlying biological mechanisms of ZIKV infection. Despite the remarkable progress that has been achieved, no drugs or vaccines have been approved for the prevention and treatment of ZIKV infection.

The ZIKV genome is comprised of three structural (C, prM, and E) and seven non-structural (NS) proteins (NS1, NS2A, NS2B, NS3, NS4A, NS4B, and NS5) [6,7]. Flaviviruses utilize diverse strategies involving NS proteins to suppress interferon (IFN)-mediated pathways. Similar to other flaviviruses, ZIKV is also capable of evading and antagonizing the host immune responses [6]. In particular, ZIKV NS1 and NS4B inhibit type I IFN pathways at the level of TANK-binding kinase, whereas NS2B and NS3 proteins inhibit Janus kinase/signal transducers and activators of transcription (JAK/STAT) signaling pathways, downstream of the type I IFN pathway, by promoting the degradation of JAK1 and suppression of virus-induced cell death [8]. In addition, our previous study has confirmed the interaction between NS1 and retinoic acid-inducible gene 1 (RIG-I) or melanoma differentiation-associated protein 5 (MDA5), and the NS1-induced inhibition of RIG-I-like receptor (RLR)-mediated IFN signaling pathways [9]. Furthermore, type I and III IFN-mediated signaling is inhibited by NS5 through its binding and degradation of STAT2 [10,11]. These findings demonstrate that ZIKV is capable of blocking IFN signaling, although details on the strategies employed by ZIKV to suppress the innate immune defense require further studies.

As negative regulators of JAK/STAT signaling, the suppressor of cytokine signaling (SOCS) family of eight proteins consist of the cytokine-inducible SH2 domain-containing protein and SOCS1–7 [12,13]. Among these proteins, SOCS1 and SOCS3 possess an amino-terminal kinase inhibitory region that inhibits JAK activity and a carboxy-terminal SOCS box that recruits the ubiquitin transferase complex. This multifaceted structure of SOCS proteins allows for a wide range of biological functions [14]. Recently, SOCS proteins have gained attention as viral targets for exploiting the host immune response. The protein expression of SOCS1 and/or SOCS3 following viral infection has been shown to promote the survival of multiple viruses [15,16,17,18,19,20,21,22]. However, further studies are necessary to delineate the primary roles and mechanisms of SOCS proteins, especially during ZIKV infection.

This study was carried out to examine the expression patterns and function of SOCS proteins during the innate immune response to ZIKV infection in various cell models, including A549 human lung adenocarcinoma cells, JAr human choriocarcinoma cells, and human neural progenitor cells (hNPCs). Upon ZIKV infection, the expression levels of SOCS1 and SOCS3 were upregulated in a manner dependent on post-infection timepoints. Our findings contribute new insights into how ZIKV utilizes the SOCS-mediated suppression of IFN for innate immune evasion.

## 2. Materials and Methods

### 2.1. Cells and Viruses

A549, JAr, and African green monkey kidney epithelial (Vero) cells obtained from the American Type Culture Collection (ATCC; Manassas, VA, USA) were used for this study. A549 and JAr cells were cultured at 37 °C in RPMI 1640 medium (Corning Mediatech, Corning, NY, USA) supplemented with 10% fetal bovine serum (FBS; Corning Mediatech) and 1% antibiotics. Vero cells were cultured at 37 °C in Dulbecco’s modified Eagle’s medium (DMEM; Corning Mediatech) supplemented with 10% FBS and 1% antibiotics. Human neural progenitor cells (hNPCs) were generated as previously described [23].

ZIKV MR766 (African origin) and PRVABC59 (Asia origin) were purchased from ATCC and propagated in Vero cells. Viral titers were determined using a standard plaque assay [9,23]. First, viral supernatants were diluted in medium and added to Vero cells seeded in 6-well plates. Viral supernatants were removed after 2 h of attachment and cells were overlaid with DMEM containing the following: 1 mg/mL bovine serum albumin; 40 mM MgCl_2_; 0.2% glucose; 2 mM sodium pyruvate; 4 mM L-glutamine, 1× pen-strep; and 0.1% NaHCO_3_. After 7 days of incubation, 10% trichloroacetic acid (Sigma-Aldrich) in PBS was used to fix the infected cells, and 0.5% crystal violet (JUNSEI, Tokyo, Japan) solution was used to stain the cells, in order to quantify the plaque-forming units.

### 2.2. Quantitative Reverse Transcription PCR (qRT-PCR) Analysis

Total RNA from cells was prepared using Trizol reagent (Invitrogen), as previously described [24]. The ImProm-II Reverse Transcription System (Promega, Madison, WI, USA) was used for first-strand cDNA synthesis from 0.5 μg of total RNA, as per the manufacturer’s instructions. Primer sequences were described previously [18,23]. The mRNA expression of genes was quantified by real-time PCR using Power SYBR^®^ Green Master Mix (Invitrogen) and was carried out using the QuantStudio 6 Flex Real-time PCR system (Thermo Fisher Scientific, Waltham, MA, USA). Real-time PCR consisted of the following steps: 95 °C for 10 min, followed by 40 cycles of 95 °C for 30 s and 60 °C for 1 min. β-actin was used as an internal reference for the normalization of gene expression quantified by real-time PCR. The data were analyzed with the conventional Ct method (2^−ΔΔCt^ method).

### 2.3. Knockdown of SOCS1 or SOCS3 Using Small Interfering RNA (siRNA)

To assess the knockdown efficiency of SOCS1 and SOCS3, transient transfection with a scrambled control, human SOCS1, or SOCS3 siRNA (Bioneer, Daejeon, Korea) was performed using Lipofectamine 2000 (Invitrogen), according to the manufacturer’s protocol. SOCS1 and SOCS3 gene expressions were quantified by qRT-PCR.

### 2.4. Confocal Microscopy

For ZIKV detection, cells were fixed with 4% formaldehyde (20 min) and permeabilized with 0.1% Triton X-100 (10 min) prior to being stained, first with the anti-pan-flavivirus envelope (E) monoclonal antibody (1:200 dilution; Abcam, Cambridge, UK) and second with the anti-mouse Alexa 594 conjugated antibody (Invitrogen). Coverslips were mounted on glass slides with mounting medium containing 4,6-diamidino-2-phenylindole (DAPI) and imaged using Zeiss LSM700 (Carl Zeiss, Oberkochen, Germany).

### 2.5. Luciferase Reporter Assay

HEK 293T cells were seeded in 96-well plates. The cells were transiently transfected with the following promoter reporter (firefly luciferase) constructs IFN-β, IFN-λ1, or IFN-λ2. IFN-λ reporter plasmids were kind gifts from Dr. Tomozumi Imamichi, Frederick National Laboratory for Cancer Research, Frederick, MD, USA [25]. The cells were transfected in combination with an empty vector (EV), RIG-I, mitochondrial antiviral signaling protein (MAVS), or TANK-binding kinase 1 (TBK1)-specific plasmid. Co-transfection with either an empty vector or SOCS plasmid was carried out to determine the effect of SOCS overexpression. As an internal control, pRL-TK plasmid was transfected simultaneously. At 24 h post-transfection, cell lysates were assessed to measure the relative luciferase activity using the luciferase reporter assay system (Promega).

### 2.6. Enzyme-Linked Immunosorbent Assay (ELISA)

Levels of IFN-λ1/3 and IFN-λ2 in cultured medium were quantified using ELISA (R&D Systems, Minneapolis, MN, USA), as per the manufacturer’s guidelines. Error bars shown in the relevant figures indicate the standard deviation of at least four independent repeats of the experiment.

### 2.7. Western Blot Analysis

The total protein was extracted with RIPA buffer (Sigma-Aldrich) and quantified using a protein reagent assay BCA kit (Thermo Scientific). Then, 8%–12% SDS-PAGE gels were used for separation, and the resolved proteins were transferred to polyvinylidene difluoride membranes (Millipore), prior to being blocked with 5% (*w*/*v*) skim milk in TBS supplemented with 0.1% (*v*/*v*) Tween-20 (TBST) for 1 h at 25 °C. Primary antibodies (Cell Signaling Technologies, Danvers, MA, USA) were incubated overnight at 4 °C. Anti-β-actin antibody was used as the normalizing control (Abgent, San Diego, CA, USA). Membranes were washed three times with TBST and incubated with the horseradish peroxidase-conjugated anti-rabbit or anti-mouse IgG secondary antibody for 1 h at 25 °C. After washing with TBST, membranes were incubated with ECL solution kit (Pierce) for visualization using the Fusion Solo Imaging System (Vilber Lourmat, Collégien, France).

### 2.8. Statistical Analysis

The results were expressed as the mean ± standard error of the mean (SEM). Student’s *t*-test was used for statistical analysis employing the GraphPad QuickCalcs software program (GraphPad Software, LaJolla, CA, USA). Differences between groups were considered significant when the *p*-value was <0.05.

## 3. Results

### 3.1. ZIKV Infection Induces SOCS1 and SOCS3 Upregulation in Various Cells

**Figure 1:**
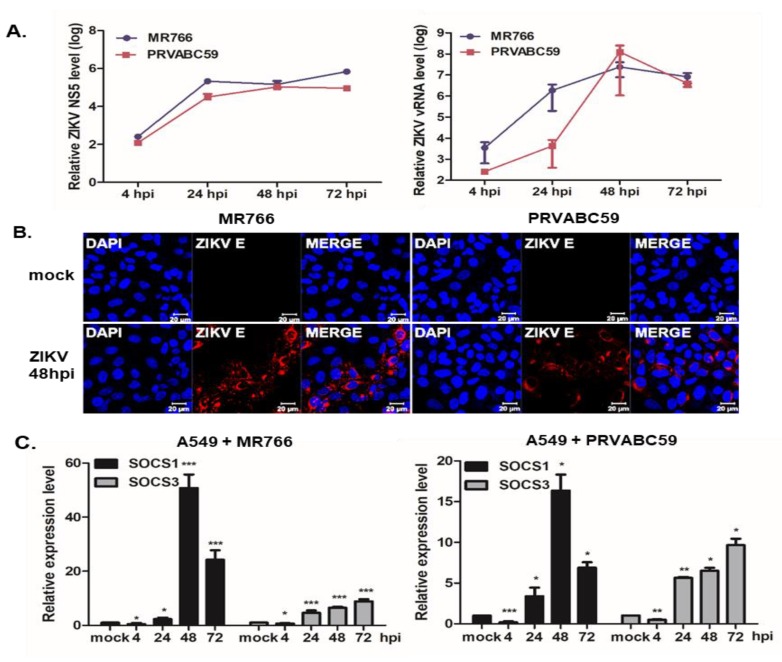
Zika virus (ZIKV) infection leads to the upregulation of suppressor of cytokine signaling (SOCS)1 and SOCS3 expression in A549 cells. Human lung adenocarcinoma (A549) cells were infected with ZIKV (MR766 or PRVABC59) at an MOI of 1 for the indicated times. (**A**) qRT-PCR was performed to measure ZIKV NS5 and (-) vRNA expression levels. (**B**) Confocal microscopy of mock- vs. ZIKV-infected cells. ZIKV E is indicated in red and nuclei are stained blue. Images are representative of three independent experiments. Scale bar represents 20 µm. (**C**) qRT-PCR was performed to measure the relative SOCS1 and SOCS3 mRNA levels following MR766 infection (left) and PRVABC59 infection (right). * *p* < 0.05; ** *p* < 0.01; *** *p* < 0.001 versus mock-infected control cells.

A549, JAr, and hNPCs were used to study the ZIKV-mediated innate immune response. A549 cells were infected at an multiplicity of infection (MOI) of 1, with either the African lineage (MR766) or Asian lineage (PRVABC59) of ZIKV. ZIKV NS5 and (-) vRNA transcript levels gradually increased in response to both MR766 and PRVABC59 infection in a time-dependent manner (Figure 1A); a finding which was consistent with our previous observation of efficient ZIKV replication in A549 cells [23]. The expression levels of ZIKV envelope (E) antigens increased in a time-dependent manner, and ZIKV E was visualized in most A549 cells at 48 h post-infection (hpi) by immunofluorescence staining (Figure 1B). The expression of SOCS1 and SOCS3 genes was quantified by qRT-PCR following MR766 or PRVABC59 infection. The suppression of both SOCS1 and SOCS3 transcript levels was observed at 4 hpi. SOCS1 transcript levels were significantly elevated upon ZIKV infection at 48 hpi, and decreased thereafter. In contrast, SOCS3 expression was upregulated following ZIKV infection at 24 hpi (Figure 1C). PRVABC59-infected A549 cells displayed similar patterns of gene expression.

**Figure 2:**
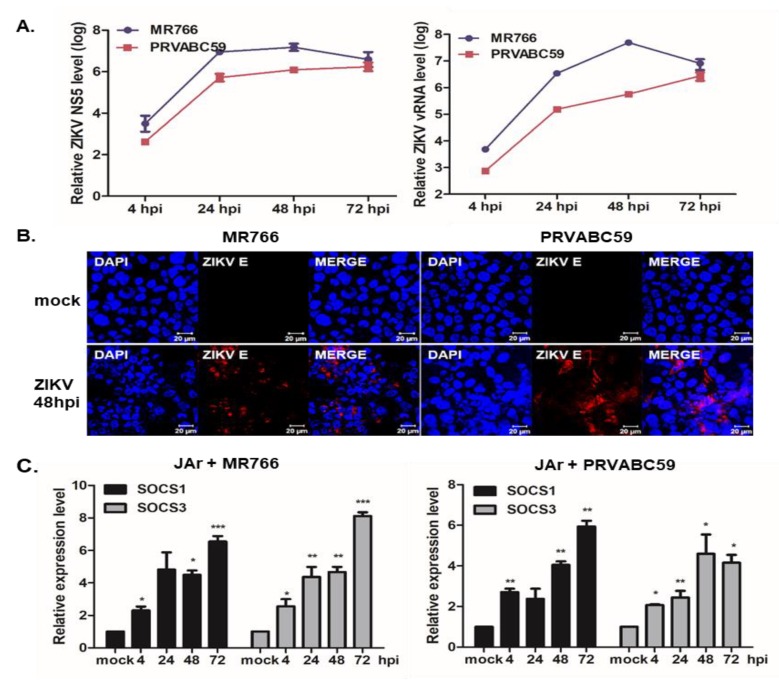
Increased SOCS1 and SOCS3 expression in ZIKV-infected JAr cells. Human choriocarcinoma JAr cells were infected with ZIKV (MR766 or PRVABC59) at an MOI of 1 for the indicated times. (**A**) qRT-PCR was performed to measure the expression levels of ZIKV NS5 and (-) vRNA. (**B**) Cells were fixed with paraformaldehyde and permeabilized with 0.5% Triton X-100. The ZIKV envelope (**E**) protein was immunostained with an anti-pan-flavivirus envelope monoclonal antibody. ZIKV E is indicated in red and cell nuclei are stained blue. The images are representative of three independent experiments. Scale bar represents 20 µm. (**C**) qRT-PCR was performed to measure the relative SOCS1 and SOCS3 mRNA levels following MR766 infection (left) and PRVABC59 infection (right). * *p* < 0.05; ** *p* < 0.01; *** *p* < 0.001 versus mock-infected control cells.

Given that ZIKV infection during early pregnancy is known to target several human placental cells, including trophoblasts, endothelial cells, fibroblasts, and Hofbauer cells [6,9], the expression patterns of SOCS proteins during ZIKV infection were examined in JAr human choriocarcinoma cells. A significant dose-dependent upregulation of ZIKV NS5 and (-) vRNA transcript levels was observed in a time-dependent manner (Figure 2A). Additionally, immunofluorescence staining of ZIKV E proteins confirmed that JAr cells were susceptible to ZIKV infection (Figure 2B). The qRT-PCR results indicated a time-dependent elevation of SOCS1 and SOCS3 mRNA expression levels in response to both MR766 and PRVABC59 infections (Figure 2C).

**Figure 3:**
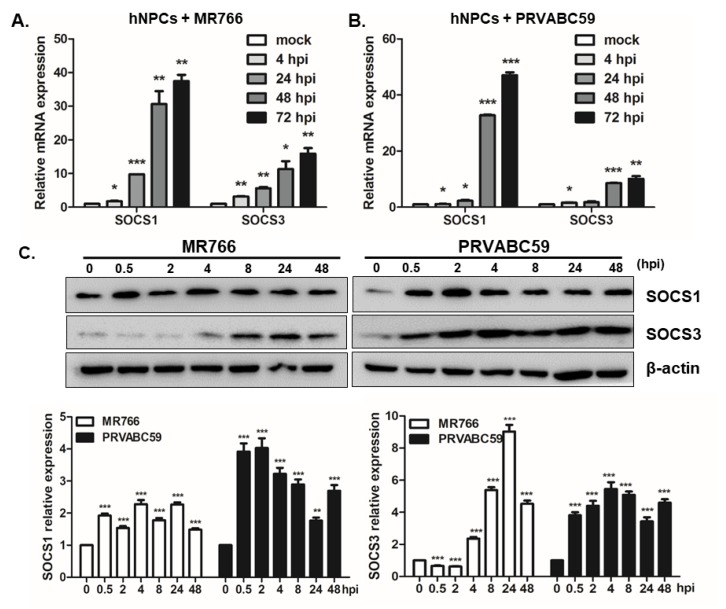
Induction of SOCS1 and SOCS3 following ZIKV infection in human neural progenitor cells (hNPCs). Human NPCs were infected with distinct lineages of ZIKV, African (MR766) (**A**) and Asian ZIKV strains (PRVABC59) (**B**), at an MOI of 1. The mRNA levels of ZIKV SOCS1 and SOCS3 were measured over time using qRT-PCR. Data are representative of three independent experiments, each performed in duplicate. Statistical analysis: * *p* < 0.05; ** *p* < 0.01; *** *p* < 0.001 versus mock-infected control cells. (**C**) The protein levels of SOCS1, SOCS3, and β-actin were analyzed by Western Blot analysis. Quantitative densitometric analysis of Western Blotting is presented, with normalized densitometric units plotted against the mock control (shown as numbers).

Previous studies utilizing various experimental models of congenital Zika syndrome identified neural progenitor cells as a primary target of viral infection [26]. Therefore, hNPCs were used to examine the expression pattern of SOCS following infection (MOI = 1) with the ZIKV African lineage (MR766) or Asian lineage (PRVABC59). As shown in Figure 3A, SOCS1 and SOCS3 expression increased at 4 hpi and continued to increase at later timepoints; a finding which indicates the early induction of SOCS expression subsequent to ZIKV infection. A further investigation was carried out to see if hNPCs display a distinct response to PRVABC59 infection. Similar patterns were observed in PRVABC59-infected hNPCs (Figure 3B). In addition, western blot results confirmed the upregulation of SOCS1 and SOCS3 protein levels triggered during the early phases of ZIKV infection (Figure 3C).

### 3.2. Overexpression or Knockdown of SOCS1 and SOCS3 Modulates ZIKV Replication

**Figure 4:**
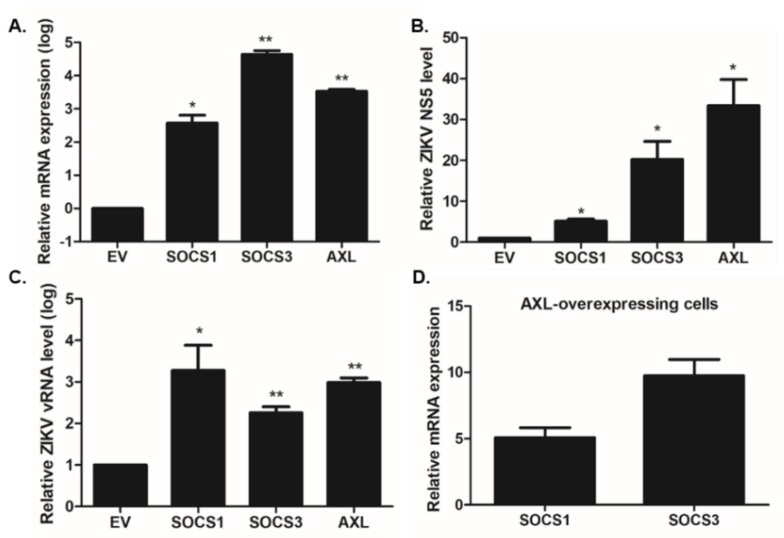
Effect of SOCS1 or SOCS3 overexpression on virus gene expression. A549 cells were transfected with an MYC-empty vector (EV), FLAG-EV, MYC-SOCS1, MYC-SOCS3, or FLAG-AXL expression plasmid for 24 h. The next day, cells were infected with ZIKV MR766 (MOI 1.0) for 24 h. (**A**) The transfection efficiency was confirmed by measuring each gene expression level. Changes in the transcriptional expression of ZIKV NS5 (**B**) or ZIKV (-) vRNA (**C**) were measured using qRT-PCR. Transcript expression levels were calculated in relation to the expression level of β-actin and expressed as a fold-change in comparison with the expression level in EV-transfected control cells. * *p* < 0.05; ** *p* < 0.01; *** *p* < 0.001 versus ZIKV-infected EV-transfected cells. (**D**) SOCS1 and SOCS3 expression levels were measured in AXL-overexpressing cells following ZIKV infection.

As ZIKV infection resulted in the upregulation of both SOCS1 and SOCS3 expression, SOCS1 or SOCS3 was overexpressed in A549 cells in order to further evaluate the functional roles of SOCS1 and SOCS3 in viral replication. Since AXL has been proposed as the entry receptor for ZIKV, AXL was used as a positive control [27]. qRT-PCR was utilized to determine the mRNA fold-induction levels of SOCS1, SOCS3, and AXL. The effect of SOCS1 and SOCS3 overexpression was validated in Figure 4A and the SOCS-mediated ZIKV replication efficiency was determined by measuring the transcript level of ZIKV NS5 or (-) vRNA. SOCS1 and SOCS3 overexpression resulted in a significant increase of ZIKV NS5 or (-) vRNA transcript levels (Figure 4B,C). Chen et al. indicated that AXL regulates the expression of SOCS1 in a STAT1/STAT2-dependent manner, thereby promoting ZIKV entry via antagonizing type I IFN signaling [28]. Therefore, we also measured the expression levels of SOCS1 and SOCS3 in AXL-overexpressing cells. Figure 4D shows increased SOCS1 and SOCS3 transcript levels following ZIKV infection AXL-overexpressing cells, compared with EV-transfected cells.

**Figure 5:**
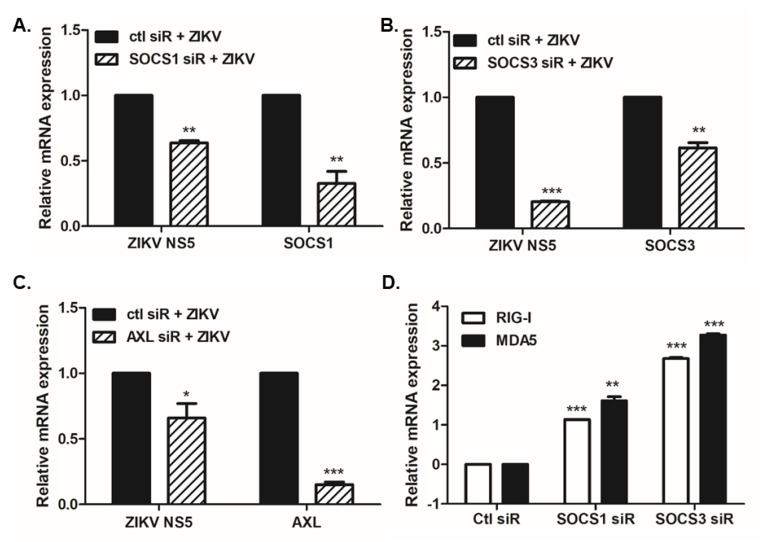
Effect of SOCS1 or SOCS3 knockdown on virus gene expression. (**A**–**C**) A549 cells were transfected with either control siRNA or SOCS1, SOCS3, or AXL siRNA. After 24 h, cells were mock-infected or infected with ZIKV MR766 (MOI 1) for 4 h. The knockdown efficiency of SOCS1, SOCS3, or AXL siRNA was determined by measuring the expression levels of SOCS1, SOCS3, or AXL mRNA, respectively. (**D**) RIG-I and MDA5 mRNA levels were determined in A549 cells transfected with either SOCS1 or SOCS3 siRNA. * *p* < 0.05; ** *p* < 0.01; *** *p* < 0.001 versus ZIKV-infected control siRNA-transfected cells.

Next, to test the knockdown effect of SOCS proteins on ZIKV replication, cells were transfected with siRNAs specific to either SOCS1 or SOCS3. After 24 h, SOCS1 and SOCS3 mRNA levels were compared to control-transfected samples. qRT-PCR revealed effective SOCS1 and SOCS3 knockdown (Figure 5). The significantly reduced levels of ZIKV NS5 expression following SOCS1 and SOCS3 knockdown highlight the importance of SOCS expression for productive viral replication.

In addition, we determined if the knockdown of SOCS1 and SOCS3 results in alterations in anti-viral gene expression. Figure 5D shows that the suppression of SOCS1 or SOCS3 expression leads to increased expression levels of cytoplasmic viral sensors, RIG-I and MDA5.

### 3.3. SOCS1 and SOCS3 Negatively Regulate Type I and III IFN Responses, Which Are Essential for ZIKV Control

**Figure 6:**
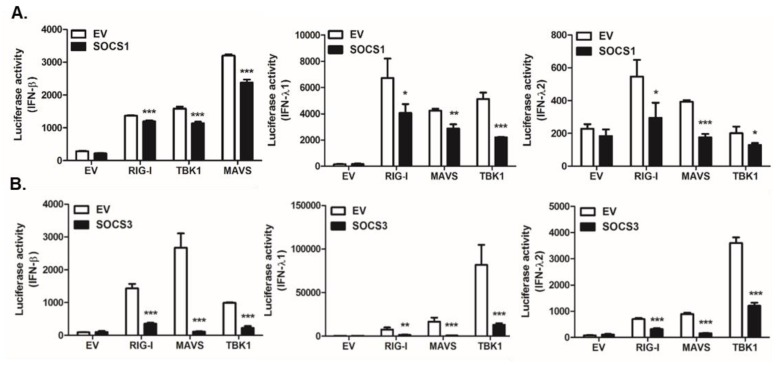
SOCS negatively regulates both type I and III interferon (IFN) pathways. HEK293T cells were transiently transfected with empty vector (EV), SOCS1 (**A**), or SOCS3 (**B**) co-transfected with the following plasmids: RIG-I, MAVS, or TBK1, along with IFN-β, IFN-λ1, and IFN-λ2 *firefly* luciferase reporter plasmids and the *Renilla* luciferase plasmid (internal control). The results are shown as the relative luciferase activity after normalization with *Renilla* luciferase activity. * *p* < 0.05; ** *p* < 0.01; *** *p* < 0.001 versus EV-transfected cells.

Several reports have shown the potential of ZIKV NS proteins to evade IFN-mediated antiviral responses via the degradation of STAT2 or targeting of TBK1 [8,9,10,11]. Next, we wanted to examine whether the induction of SOCS proteins modulates IFN pathways. A dual-luciferase reporter assay was performed using HEK293T cells transfected with plasmids carrying the IFN-β, IFN-λ1, or IFN-λ2 promoter luciferase reporter; an internal control Renilla luciferase; and RIG-I, MAVS, or TBK1 plasmids in the absence or presence of the SOCS-expressing vector. SOCS1 or SOCS3 overexpression significantly attenuated the activation of IFN-β, IFN-λ1, and IFN-λ2 promoters when induced by RIG-I, MAVS, or TBK1 plasmid transfection (Figure 6).

**Figure 7:**
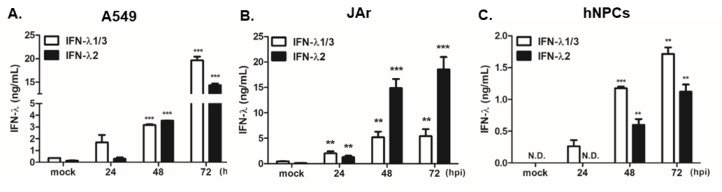
Type III IFN production is induced by ZIKV infection. A549 human lung adenocarcinoma cells (**A**), JAr human choriocarcinoma cells (**B**), and human neural progenitor cells (hNPCs) (**C**) were infected with ZIKV MR766 at an MOI of 1. The concentration of secreted IFN-λ1/3 and IFN-λ2 in cultured medium was determined by ELISA. * *p* < 0.05; ** *p* < 0.01; *** *p* < 0.001 versus mock-infected control cells.

Given the protective role of IFN-λ against ZIKV in the placenta [29], we wanted to determine if ZIKV infection results in the modulation of type III IFN production in cells that upregulate SOCS expression. A549 cells, JAr cells, and hNPCs were infected with ZIKV, and the secretion level of IFN was measured at 24, 48, and 72 hpi by ELISA. A significant increase in both IFN-λ1/3 and IFN-λ2 in response to ZIKV infection was evident in all cell types. In particular, A549 and JAr cells secreted high levels of both IFN-λ1/3 and IFN-λ2 (Figure 7).

## 4. Discussion

ZIKV, a member of the *Flaviviridae* family, has recently emerged as an important human pathogen due to its association with severe complications in newborns. Several distinct features of the mechanism utilized by ZIKV to evade the innate immune responses also allow the virus to establish an effective infection. For example, NS proteins of ZIKV act as potent inhibitors of IFN signaling pathways [8,9,10,11]. In this study, we investigated the pattern of SOCS expression in various cells in response to infection with different lineages of ZIKV. Here, we provide a new understanding of how ZIKV infection induces SOCS gene expression in order to modulate viral replication via the suppression of type I and III IFNs.

The induction of SOCS proteins is one of the various molecular mechanisms of immune evasion that allow viruses to suppress the host immunity. Notably, influenza virus studies best describe this inhibitory role of SOCS proteins, among which SOCS1 and SOCS3 are critical regulators of influenza A virus-triggered innate immune responses [21,30,31,32]. Influenza A virus has also been reported to inhibit type I IFN signaling through the induction of SOCS3 expression for an impaired antiviral response [20]. According to this study, SOCS may be involved in the suppression of type I IFN signaling at the level of JAK/STAT activation by influenza A viruses. Moreover, members of the SOCS family are induced by infections with various viruses, including human immunodeficiency virus-1, hepatitis C virus, hepatitis B virus, herpes simplex virus type 1, respiratory syncytial virus, Ebola virus, and varicella zoster virus, and subsequently contribute to viral replication and pathogenesis [14,15,16,18,19,33,34,35,36].

Only a few studies have examined the involvement of SOCS during flavivirus infection. For example, dengue virus infection was reported to drive IL-10-mediated SOCS3 expression and the consequent inactivation of JAK/STAT pathways [37]. Additionally, a close association between cytokine imbalance and SOCS1/3 observed in patients with a severe dengue infection suggested that the combined analysis of SOCS1/3, IL-10, and IL-6 expression levels could identify at-risk patients for severe dengue [38]. In an intracerebral infection mouse model, SOCS proteins were upregulated in brain resident cells in response to infection with yellow fever virus [39]. Moreover, Japanese encephalitis virus infection in mice led to an upregulation of SOCS1 and SOCS3 in vitro and in vivo in the mouse brain [17]. Our study presents new findings for the modulation of viral replication by ZIKV-induced expression of the SOCS protein. Recently, the transcriptomic analysis of ZIKV-infected dendritic cells in Sun et al. emphasized SOCS3 as one of most upregulated genes and a likely key player in the observed downregulation of interferon-stimulated genes (ISGs) [40]. Additionally, Dhiman et al. reported an increased SOCS expression upon ZIKV infection in human Schwann cells [41]. In accordance with these data, we also observed that the knockdown of SOCS led to an enhanced expression of RIG-I and MDA5, suggesting that SOCS is a negative regulator of ISGs. Therefore, it will be interesting to further research the expression and role of SOCS in various cell types susceptible to ZIKV infection.

Lineage-dependent differences have been described in the progression of ZIKV infection [42]. While both African (MR766) and Asian (PRVABC59) lineages replicated most efficiently in human epidermal keratinocytes, the MR766 strain had a higher replication efficiency in hNPCs in comparison to PRVABC59, as reported in our previous study [9]. In the current study, both SOCS1 and SOCS3 transcript levels were significantly elevated upon ZIKV infection, and the induction levels were similar for the African and Asian lineages. Interestingly, among the three cell types, hNPCs, specifically, showed the highest mRNA expression levels of SOCS1 and SOCS3. For this reason, it is highly plausible that the high infectivity of ZIKV in hNPCs has contributed to the corresponding increase in SOCS protein expression.

A recent study reported that SOCS3 inhibits IFN-β signaling pathways by promoting the ubiquitination and degradation of TBK1 [43]. Meanwhile, another study suggested that SOCS1 negatively regulates IFN-λ signaling pathways and IFN-λ-induced ISGs [44]. Our data also confirmed that the overexpression of either SOCS1 or SOCS3 led to the inhibition of both IFN-β and IFN-λ signaling pathways. The role of type III IFNs has been studied extensively in the context of ZIKV infection. In cells of epithelial origin, IFN-λ is an important type of IFN that mounts an antiviral response. For example, IFN-λ produced by human placental trophoblasts can protect against a ZIKV infection [29]. IFN-λ treatment in vivo reportedly diminished the extent of ZIKV infection in human mid-gestation fetal- and maternal-derived tissue explant models, suggesting the therapeutic potential of IFN-λ as an immunomodulator [45]. In another study, fetus-driven IFN-λ signaling contributed to antiviral responses in multiple fetal organs, including the brain [46]. Likewise, the therapeutic administration of IFN-λ1 in ZIKV-infected pregnant mice inhibited the viral burden in both maternal and fetal organs, suggesting the possibility of employing IFN-λ1 as a potential therapeutic for ZIKV-associated diseases [46]. Our data, suggesting increased IFN-λ secretions upon ZIKV infection from hNPCs, differs from the results of the transcriptomic analysis of ZIKV (MR766)-infected hNPCs in Tang et al., which did not report a significant increase in IFN-λ expression [47]. This difference may have arisen from the differences in the time following infection at which the analysis was carried out. Tang et al. analyzed the gene expression profile at 56 hpi and organized a list of up- or downregulated DEGs, but the study did not provide any validation of the data obtained using qRT-PCR. Given that our data shows the suppression of type I and III IFN signaling by a mechanism involving SOCS expression, this observation of a new aspect adds to the current knowledge of evasion strategies used by ZIKV to antagonize antiviral responses.

In conclusion, our data reveal that the ZIKV-induced expression of SOCS1/3 can effectively countermeasure the innate immune response, and suggest that type I and III IFN signaling may be targets of SOCS-mediated immune suppression. Interestingly, SOCS1 promoter variants are associated with hepatitis B virus susceptibility [48], and SOCS3 expression and genetic polymorphism influence the pathogenesis and outcome of antiviral treatment in hepatitis C virus-infected patients [49]. Provided that the recently developed SOCS1/3 antagonist has a potent antiviral function against a broad group of viruses, such as herpes simplex virus-1, vaccinia virus, and influenza virus [50,51], it will be interesting to evaluate the effect of these antagonists against ZIKV infection. The findings of this study accompanied by prospective experimental results will better advance our comprehensive understanding of the pathogenesis of ZIKV-associated diseases.

## References

[1] Dick G.W., Kitchen S.F., Haddow A.J. (1952). Zika virus. I. Isolations and serological specificity. Trans. R. Soc. Trop. Med. Hyg..

[2] Leal M.C., Muniz L.F., Ferreira T.S., Santos C.M., Almeida L.C., Van Der Linden V., Ramos R.C., Rodrigues L.C., Neto S.S. (2016). Hearing loss in infants with microcephaly and evidence of congenital zika virus infection—Brazil, November 2015–May 2016. Morb. Mortal. Wkly. Rep..

[3] Cao-Lormeau V.M., Blake A., Mons S., Lastere S., Roche C., Vanhomwegen J., Dub T., Baudouin L., Teissier A., Larre P. (2016). Guillain-barre syndrome outbreak associated with Zika virus infection in French polynesia: A case-control study. Lancet.

[4] Araujo L.M., Ferreira M.L., Nascimento O.J. (2016). Guillain-barre syndrome associated with the Zika virus outbreak in Brazil. Arq. Neuro-Psiquiatr..

[5] Lazear H.M., Diamond M.S. (2016). Zika virus: New clinical syndromes and its emergence in the western hemisphere. J. Virol..

[6] Lee J.K., Shin O.S. (2019). Advances in Zika virus-host cell interaction: Current knowledge and future perspectives. Int. J. Mol. Sci..

[7] Elshahawi H., Syed Hassan S., Balasubramaniam V. (2019). Importance of Zika virus NS5 protein for viral replication. Pathogens.

[8] Wu Y., Liu Q., Zhou J., Xie W., Chen C., Wang Z., Yang H., Cui J. (2017). Zika virus evades interferon-mediated antiviral response through the co-operation of multiple nonstructural proteins in vitro. Cell Discov..

[9] Kim J.A., Seong R.K., Son S.W., Shin O.S. (2019). Insights into ZIKV-mediated innate immune responses in human dermal fibroblasts and epidermal keratinocytes. J. Investig. Dermatol..

[10] Kumar A., Hou S., Airo A.M., Limonta D., Mancinelli V., Branton W., Power C., Hobman T.C. (2016). Zika virus inhibits type-I interferon production and downstream signaling. EMBO Rep..

[11] Grant A., Ponia S.S., Tripathi S., Balasubramaniam V., Miorin L., Sourisseau M., Schwarz M.C., Sanchez-Seco M.P., Evans M.J., Best S.M. (2016). Zika virus targets human STAT2 to inhibit type I interferon signaling. Cell Host Microbe.

[12] Yoshimura A., Naka T., Kubo M. (2007). SOCS proteins, cytokine signalling and immune regulation. Nat. Rev. Immunol..

[13] Hilton D.J., Richardson R.T., Alexander W.S., Viney E.M., Willson T.A., Sprigg N.S., Starr R., Nicholson S.E., Metcalf D., Nicola N.A. (1998). Twenty proteins containing a C-terminal SOCS box form five structural classes. Proc. Natl. Acad. Sci. USA.

[14] Akhtar L.N., Benveniste E.N. (2011). Viral exploitation of host SOCS protein functions. J. Virol..

[15] Akhtar L.N., Qin H., Muldowney M.T., Yanagisawa L.L., Kutsch O., Clements J.E., Benveniste E.N. (2010). Suppressor of cytokine signaling 3 inhibits antiviral IFN-beta signaling to enhance HIV-1 replication in macrophages. J. Immunol..

[16] Shao R.X., Zhang L., Hong Z., Goto K., Cheng D., Chen W.C., Jilg N., Kumthip K., Fusco D.N., Peng L.F. (2013). SOCS1 abrogates IFN’s antiviral effect on hepatitis C virus replication. Antivir. Res..

[17] Li X., Zhu Q., Cao Q., Chen H., Qian P. (2014). Japanese encephalitis virus upregulates the expression of SOCS3 in mouse brain and Raw264.7 cells. Viruses.

[18] Choi E.J., Lee C.H., Shin O.S. (2015). Suppressor of cytokine signaling 3 expression induced by varicella-zoster virus infection results in the modulation of virus replication. Scand. J. Immunol..

[19] Okumura A., Rasmussen A.L., Halfmann P., Feldmann F., Yoshimura A., Feldmann H., Kawaoka Y., Harty R.N., Katze M.G. (2015). Suppressor of cytokine signaling 3 is an inducible host factor that regulates virus egress during ebola virus infection. J. Virol..

[20] Pauli E.K., Schmolke M., Wolff T., Viemann D., Roth J., Bode J.G., Ludwig S. (2008). Influenza A virus inhibits type I IFN signaling via NF-kappaB-dependent induction of SOCS-3 expression. PLoS Pathog..

[21] Pothlichet J., Chignard M., Si-Tahar M. (2008). Cutting edge: Innate immune response triggered by influenza A virus is negatively regulated by SOCS1 and SOCS3 through a RIG-I/IFNAR1-dependent pathway. J. Immunol..

[22] Sun K., Salmon S., Yajjala V.K., Bauer C., Metzger D.W. (2014). Expression of suppressor of cytokine signaling 1 (SOCS1) impairs viral clearance and exacerbates lung injury during influenza infection. PLoS Pathog..

[23] Kim J.A., Seong R.K., Kumar M., Shin O.S. (2018). Favipiravir and ribavirin inhibit replication of Asian and African strains of Zika virus in different cell models. Viruses.

[24] Oh S.J., Lim S., Song M.J., Ahn J.H., Lee C.H., Shin O.S. (2019). Whole transcriptome analyses reveal differential mRNA and microRNA expression profiles in primary human dermal fibroblasts infected with clinical or vaccine strains of Varicella Zoster virus. Pathogens.

[25] Sui H., Zhou M., Chen Q., Lane H.C., Imamichi T. (2014). siRNA enhances DNA-mediated interferon lambda-1 response through crosstalk between RIG-I and IFI16 signalling pathway. Nucleic Acids Res..

[26] Miner J.J., Diamond M.S. (2016). Understanding how Zika virus enters and infects neural target cells. Cell Stem Cell.

[27] Nowakowski T.J., Pollen A.A., Di Lullo E., Sandoval-Espinosa C., Bershteyn M., Kriegstein A.R. (2016). Expression analysis highlights AXL as a candidate Zika virus entry receptor in neural stem cells. Cell Stem Cell.

[28] Chen J., Yang Y.F., Yang Y., Zou P., Chen J., He Y., Shui S.L., Cui Y.R., Bai R., Liang Y.J. (2018). AXL promotes Zika virus infection in astrocytes by antagonizing type I interferon signalling. Nat. Microbiol..

[29] Bayer A., Lennemann N.J., Ouyang Y., Bramley J.C., Morosky S., Marques E.T., Cherry S., Sadovsky Y., Coyne C.B. (2016). Type III interferons produced by human placental trophoblasts confer protection against Zika virus infection. Cell Host Microbe.

[30] Ye S., Lowther S., Stambas J. (2015). Inhibition of reactive oxygen species production ameliorates inflammation induced by influenza A viruses via upregulation of SOCS1 and SOCS3. J. Virol..

[31] Wei H., Wang S., Chen Q., Chen Y., Chi X., Zhang L., Huang S., Gao G.F., Chen J.L. (2014). Suppression of interferon lambda signaling by SOCS-1 results in their excessive production during influenza virus infection. PLoS Pathog..

[32] Ramirez-Martinez G., Cruz-Lagunas A., Jimenez-Alvarez L., Espinosa E., Ortiz-Quintero B., Santos-Mendoza T., Herrera M.T., Canche-Pool E., Mendoza C., Banales J.L. (2013). Seasonal and pandemic influenza H1N1 viruses induce differential expression of SOCS-1 and RIG-I genes and cytokine/chemokine production in macrophages. Cytokine.

[33] Reichard A.C., Cheemarla N.R., Bigley N.J. (2015). SOCS1/3 expression levels in HSV-1-infected, cytokine-polarized and -unpolarized macrophages. J. Interferon Cytokine Res..

[34] Hashimoto K., Ishibashi K., Ishioka K., Zhao D., Sato M., Ohara S., Abe Y., Kawasaki Y., Sato Y., Yokota S. (2009). RSV replication is attenuated by counteracting expression of the suppressor of cytokine signaling (SOCS) molecules. Virology.

[35] Kim K.A., Lin W., Tai A.W., Shao R.X., Weinberg E., De Sa Borges C.B., Bhan A.K., Zheng H., Kamegaya Y., Chung R.T. (2009). Hepatic SOCS3 expression is strongly associated with non-response to therapy and race in HCV and HCV/HIV infection. J. Hepatol..

[36] Sood V., Lata S., Ramachandran V.G., Banerjea A.C. (2019). Suppressor of cytokine signaling 3 (SOCS3) degrades p65 and regulate HIV-1 replication. Front. Microbiol..

[37] Ubol S., Phuklia W., Kalayanarooj S., Modhiran N. (2010). Mechanisms of immune evasion induced by a complex of dengue virus and preexisting enhancing antibodies. J. Infect. Dis..

[38] Flores-Mendoza L.K., Estrada-Jimenez T., Sedeno-Monge V., Moreno M., Manjarrez M.D.C., Gonzalez-Ochoa G., Millan-Perez Pena L., Reyes-Leyva J. (2017). IL-10 and socs3 are predictive biomarkers of dengue hemorrhagic fever. Mediat. Inflamm..

[39] Steffensen M.A., Fenger C., Christensen J.E., Jorgensen C.K., Bassi M.R., Christensen J.P., Finsen B., Thomsen A.R. (2014). Suppressors of cytokine signaling 1 and 3 are upregulated in brain resident cells in response to virus-induced inflammation of the central nervous system via at least two distinctive pathways. J. Virol..

[40] Sun X., Hua S., Chen H.R., Ouyang Z., Einkauf K., Tse S., Ard K., Ciaranello A., Yawetz S., Sax P. (2017). Transcriptional changes during naturally acquired Zika virus infection render dendritic cells highly conducive to viral replication. Cell Rep..

[41] Dhiman G., Abraham R., Griffin D.E. (2019). Human schwann cells are susceptible to infection with Zika and yellow fever viruses, but not dengue virus. Sci. Rep..

[42] Dowall S.D., Graham V.A., Rayner E., Hunter L., Atkinson B., Pearson G., Dennis M., Hewson R. (2017). Lineage-dependent differences in the disease progression of Zika virus infection in type-I interferon receptor knockout (A129) mice. PLoS Negl. Trop. Dis..

[43] Liu D., Sheng C., Gao S., Yao C., Li J., Jiang W., Chen H., Wu J., Pan C., Chen S. (2015). SOCS3 drives proteasomal degradation of TBK1 and negatively regulates antiviral innate immunity. Mol. Cell Biol..

[44] Blumer T., Coto-Llerena M., Duong F.H.T., Heim M.H. (2017). SOCS1 is an inducible negative regulator of interferon lambda (IFN-lambda)-induced gene expression in vivo. J. Biol. Chem..

[45] Jagger B.W., Miner J.J., Cao B., Arora N., Smith A.M., Kovacs A., Mysorekar I.U., Coyne C.B., Diamond M.S. (2017). Gestational stage and IFN-lambda signaling regulate ZIKV infection in utero. Cell Host Microbe.

[46] Chen J., Liang Y., Yi P., Xu L., Hawkins H.K., Rossi S.L., Soong L., Cai J., Menon R., Sun J. (2017). Outcomes of congenital Zika disease depend on timing of infection and maternal-fetal interferon action. Cell Rep..

[47] Tang H., Hammack C., Ogden S.C., Wen Z., Qian X., Li Y., Yao B., Shin J., Zhang F., Lee E.M. (2016). Zika virus infects human cortical neural progenitors and attenuates their growth. Cell Stem Cell.

[48] Zhang P., Li F., Li N., Zhu Q., Yang C., Han Q., Chen J., Lv Y., Yu L., Wei P. (2014). Genetic variations of SOCS1 are associated with chronic hepatitis B virus infection. Hum. Immunol..

[49] Aslam R., Raza S.M., Naeemi H., Mubarak B., Afzal N., Khaliq S. (2016). SOCS3 mRNA expression and polymorphisms as pretreatment predictor of response to HCV genotype 3A IFN-based treatment. SpringerPlus.

[50] Ahmed C.M., Dabelic R., Martin J.P., Jager L.D., Haider S.M., Johnson H.M. (2010). Enhancement of antiviral immunity by small molecule antagonist of suppressor of cytokine signaling. J. Immunol..

[51] Ahmed C.M., Dabelic R., Bedoya S.K., Larkin J., Johnson H.M. (2015). A SOCS1/3 antagonist peptide protects mice against lethal infection with influenza a virus. Front. Immunol..

